# Characterization and Phylogenetic Implications of the Complete Mitochondrial Genome of Syrphidae

**DOI:** 10.3390/genes10080563

**Published:** 2019-07-25

**Authors:** Hu Li

**Affiliations:** 1 Shaanxi Key Laboratory of Bio-Resources, School of Biological Science & Engineering, Shaanxi University of Technology, Hanzhong 723000, China; lihu@snut.edu.cn; Tel.: +86-158-0916-3306; 2 College of Life Sciences, Northwest University, Xi’an 710069, China

**Keywords:** Syrphidae, Muscomorpha, mitogenome, phylogeny

## Abstract

In this study, the complete mitochondrial genomes (mitogenomes) of two hoverfly species of *Korinchia angustiabdomena* (Huo, Ren, and Zheng) and *Volucella nigricans* Coquillett (Diptera: Syrphidae) were determined and analyzed. The circular mitogenomes were 16,473 bp in *K. angustiabdomena* (GenBank No. MK870078) and 15,724 bp in *V. nigricans* (GenBank No. MK870079). Two newly sequenced mitogenomes both contained 37 genes, and the gene order was similar with other syrphine species. All the protein-coding genes (PCGs) were started with the standard ATN codons; and most of PCGs were terminated with a TAA stop codon, while *ND1* in *K. angustiabdomena* ended with a TAG codon, and *ND5* terminated with truncated T stop codons in both species. The phylogenetic relationship between *K. angustiabdomena* and *V. nigricans* with related lineages was reconstructed using Bayesian inference and Maximum-likelihood analyses. The monophyly of each family considered within Muscomorpha was confirmed by the clades in the phylogenetic tree, and superfamily of the Oestroidea (Calliphoridae, Sarcophagidae, and Oestridae) was unexpectedly found to be a paraphyletic group based on our selected data. This mitogenome information for *K. angustiabdomena* and *V. nigricans* could facilitate future studies of evolutionarily related insects.

## 1. Introduction

Syrphidae, true flies, is one group in the very large insect order Diptera. Syrphidae are well known by the common names flower flies or hoverflies. They can be found hovering around flowers and are often mistaken for bees or wasps due to their black and yellow coloring. Syrphidae can be recognized from its vena spuria on the wing. It is cosmopolitan and includes more than 6,000 species known around the world [1,2,3].

While the adult Syrphidae typically visits flowers, their larvae are often polyphagous in a very diverse habitat. The larvae of subfamily Syrphinae are largely predaceous usually on soft-bodied Hemiptera [4], typically aphids. There are also some non-predaceous Syrphinae that develop as miners in plants [4,5,6]; Eristalinae are generally saprophagous in dead wood; coprophagous, phytophagous, aquatic filter feeders, or inquiline in social insect nests [2,7]; Microdontinae inquiline with ants in nests.

In general, Syrphidae were always classified into three groups (Microdontinae, Eristalinae, and Syrphinae) based on adult morphology [2,8]. There is some controversy over the classification of Pipizinae, which share a larval feeding mode with Syrphinae but some external morphological characters with the Eristalinae. Recently, Mengual et al. proposed that Pipizinae be upgraded to the level of subfamily based on morphological and genomic data [4].

Recent studies based on integrated datasets almost unanimously agree that the Microdontinae was a sister group to the remaining syrphines; Pipizinae and Syrphinae formed one clade, and Eristalinae was inferred to be paraphyletic [4,9,10,11]. More importantly, the monophyletic Syrphoidea was still not supported by a recent phylogenomics study using larger-scale transcriptomic data [11].

The animal mitochondrial genome, a typical circular DNA molecule, is approximately 16 kb long and consists of 37 genes including 13 protein-coding genes (PCGs), two ribosomal RNA (rRNA) genes, and 22 transfer RNA (tRNA) genes, plus an A + T-rich region [12,13,14]. It has a small genome size, conserved gene content and organization, lack of extensive recombination, maternal inheritance, and a high nucleotide substitution rate relative to the nuclear genomes, providing higher phylogenetic resolution than short sequences of single genes in many insects [15,16,17,18,19].

Up to now, the sequencing of mitochondrial genomes from only six syrphines have been reported in GenBank and the literature. Of these, *Simosyrphus grandicornis* (accession No.: NC_008754) [20], *Episyrphus balteatus* (accession No.: NC_036481) [14], *Eupeodes corolla* (accession No.: NC_036482) [14], and *Eristalis tenax* (accession No.: NC_041143) [21] are complete, and *Syrphidae* sp. (accession No.: KM244713.1) [22]) and *Ocyptamus sativus* (accession No.: KT272862.1) [23] are partial.

*Volucella nigricans* Coquillett, 1898 (Syrphidae: Eristalinae: Volucellini), a large hoverfly, wasp mimic, has a large black pattern on the dorsal thorax and abdomen (with white or pale-yellow markings on segment 2 of the abdomen) and strong black wing clouds. *Korinchia angustiabdomena* (Huo, Ren and Zheng, 2007) [1] (Eristalinae: Milesiini) mimics a medium-sized bee with a black body, rather narrowed abdomen, and white hairs on lateral margins of thorax. Both hoverflies can be easily identified and collected in the field and were selected as our material.

To enrich the mitogenomes of Syrphidae and try to discuss evolutionary relationships of Syrphidae and related groups, the present paper sequenced the complete mitogenomes of these two species, uploaded both whole complete sequences to GenBank, and obtained their accession numbers of MK870078 and MK870079. Then, we compared and analyzed the phylogenetic relationships among them and other syrphine species.

## 2. Material and Methods

### 2.1. Sample Collection and DNA Extraction

Syrphine specimens used in this study were collected by sweep net and immediately preserved in absolute ethyl alcohol for the next experiment. Their collection information is provided in Table S1. Voucher specimens were deposited in the Institute of Entomology, Guizhou University in Guiyang, China. The samples were washed twice by vortexing them in absolute ethanol, and then, they were dried at room temperature before DNA extraction. Genomic DNA was extracted using a DNeasy^©^ Tissue Kit (Qiagen, Hilden, Germany). Specimens were incubated at 56 °C for 6 h to lyse cells completely, and the total genomic DNA was eluted with 100 μL of double-distilled water (ddH_2_O), while the remaining steps were conducted in accordance with the manufacturer’s protocol. The genomic DNA concentration was quantified by a system of NanoDrop 1000 and then stored at −20 °C.

### 2.2. PCR Amplification, Cloning, and Sequencing

Mitogenomes were sequenced by next-generation sequencing (Illumina HiSeq 2500, paired-end strategy and 2 Gb raw data; Berry Genomic, Beijing, China) and PCR amplification. Two sequence fragments of *COX1* (700 bp) and *12S* rRNA (550 bp) were amplified by PCR amplification as “reference sequences” using universal primers (Table 1). PCR amplifications were conducted using PCR MasterMix (Tiangen Biotech Co., Ltd., Beijing, China) according to the specification manual. The PCR cycling conditions comprised a pre-denaturation at 94 °C for 3 min and 30 cycles of denaturation at 94 °C for 30 s, annealing at a suitable temperature for 30 s and elongation at 70 °C for 1 min and an additional elongation step at 70 °C for 10 min at the end of all cycles. The sequencing results obtained from PCR amplification and TA cloning were assembled using the SeqMan program package (DNAStar Inc.; Madison, WI, USA) (Figure S1). Clean next-generation sequencing results were assembled using Geneious R9 [24] based on the *COX1* and *12S* fragment of mitochondrial DNA, A total of 60,031,528 reads were generated and 294,173 were assembled in *K. angustiabdomena* and, respectively, 48,244,868 and 287,730 in *V. nigricans*.

### 2.3. Mitochondrial Genome Annotation

The mitochondrial genome (mitogenome) was initially annotated using the MITOS web server [27]. The base composition was analyzed with MEGA 6.0 [28], and PCGs were identified in GenBank [29]. The locations and secondary structures of 22 tRNA genes were determined using tRNA scan-SE version 1.21 [30] and ARWEN version 1.2 [31]. The rRNA genes were determined based on the locations of adjacent tRNA genes and by comparisons with other Syrphoidea. DNASIS version 2.5 (Hitachi Engineering, Tokyo, Japan) and RNA Structure version 5.2 [32] were used to predict helical elements in variable regions. Strand asymmetry was calculated using the formula: AT skew = (A − T)/(A + T) and GC skew = (G − C)/(G + C) [33].

### 2.4. Sequence Alignment and Phylogenetic Analysis

A total of 42 insect species were used in the phylogenetic analysis, including 40 ingroup species and 2 outgroup species (*Cydistomyia duplonotata* and *Trichophthalma punctate* [20]). The ingroups included 11 families all which have representative mitogenome sequences in Diptera (Table S2). The 13 PCG sequences without stop codons were used in the phylogenetic analysis. Each PCG was aligned individually with codon-based multiple alignments using the MAFFT algorithm in the Translator X online server [34] with gaps and ambiguous sites removed from the protein alignment before back-translating to nucleotides using Gblocks under the default settings. Next, all alignments were checked and corrected manually in MEGA 6.0 [28].

Three datasets were generated: (1) amino acid sequences of 13 PCGs with 3,681 amino acids (AA); (2) 13 PCGs and 2 rRNAs with 13,091 nucleotides (PCGRNA); and (3) the first and second codon positions of the 13 PCGs and 2 rRNAs with 9,410 nucleotides. The optimal partition scheme for each dataset and the best model for each partition were selected under the corrected Bayesian Information Criterion using Partition Finder 2 (Table S3) [35]; maximum-likelihood (ML) phylogenetic trees were constructed with the IQ-TREE using an ultrafast bootstrap approximation approach with 10,000 replicates [36]. Bayesian analyses were carried out with the site-heterogeneous model CAT + GTR implemented in PhyloBayes (PB) MPI on XSEDE [37,38].

## 3. Results and Discussion

### 3.1. Genome Organization and Base Composition

**Figure 1:**
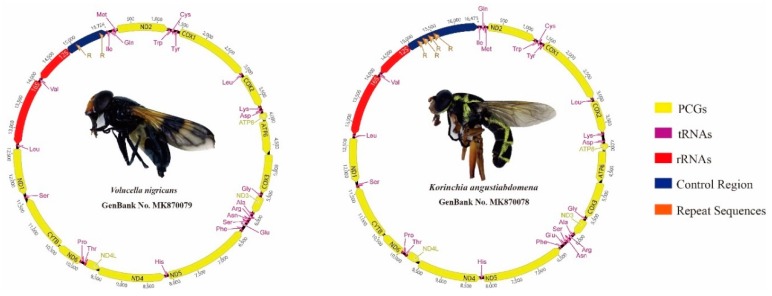
Mitochondrial genomes of two sequenced hoverflies.

**Figure 2:**
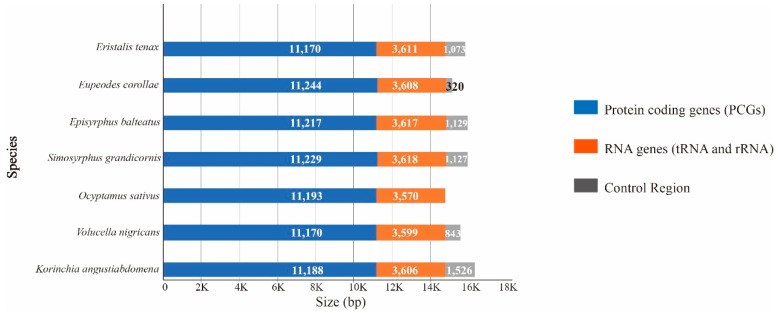
The size of PCGs, RNAs, and control regions, respectively, among the sequenced Syrphidae mitogenomes.

In this study, two complete mitochondrial genomes (mitogenomes) of Syrphidae were sequenced and annotated for the first time (Figure 1). Each newly sequenced mitogenome is circular and double stranded, containing 37 mitochondrial genes (13 PCGs, 22 tRNAs and 2 rRNAs) and one control region (Tables S4 and S5). The sizes of the two mitogenomes were 16,473 bp in *K. angustiabdomena* (GenBank No. MK870078) and 15,724 bp in *V. nigricans* (GenBank No. MK870079), respectively. Thus, Syrphidae mitogenomes range from 15,326 bp (*E. corollae*; NC_036482 [14]) to 16,473 bp (*K. angustiabdomena*, MK870078, this study). Within syrphine mitogenomes, length variation is limited in the PCGs and RNAs, but there is remarkable variation in the size of the control region (Figure 2). The patterns of mitogenome genes in the newly sequenced species are the same as those found in all previously sequenced Syrphidae, as well as of the inferred most insect mitogenome order [16]. A total of 22 genes (9 PCGs and 13 tRNAs) were encoded on the majority strand (J-strand), whereas the remaining 15 genes (4 PCGs, 9 tRNAs, and 2 rRNAs) were located on the minority strand (N strand) (Figure 1, Tables S4 and S5).

The nucleotide composition of the seven sequenced mitogenome sequences showed biases toward A and T, with the overall A + T content of the mitogenomes ranging from 79.9% (*V. nigricans*) to 80.9% (*S. grandicornis* [20]). The A + T content of the control region (mean value = 93.18%) was always higher than in other regions, while PCGs showed the lowest A + T content values (mean value = 78.44%). All mitogenomes showed positive AT skews (0.00 in *S. grandicornis* and *E. balteatus* to 0.05 in *V. nigricans*) and negative GC skews (−0.21 in *V. nigricans* to −0.13 in *E. balteatus* and *E. corollae*).

### 3.2. Protein-Coding Genes and Codon Usage

The length of PCGs are 11,188 bp in *K. angustiabdomena* and 11,170 bp in *V. nigricans* respectively, their locations and directions of 13 PCGs are similar to other syrphine. The overall A + T content of the 13 PCGs in the seven species was between 77.6% (*K. angustiabdomena*) and 79% (*O. sativus* [23]). The AT skews were slightly negative from −0.15 (*K. angustiabdomena*) to −0.12 (*V. nigricans*); the AT skews of the other five species were −0.14. The GC skews were slightly positive from 0.01 (*V. nigricans*) to 0.05 (*O. sativus*), expect in *K. angustiabdomena* (−0.01) (Table 2).

In the two newly sequenced mitogenomes, all the PCGs started with the standard ATN codons. Most PCGs terminated with a TAA stop codon, while *ND1* in *K. angustiabdomena* ended with a TAG codon and *ND5* terminated with truncated T stop codons in both species. Comparing with other Syrphidae mitogenomes, most PCGs use canonical start codons. *E. tenax* is excluded with *ND4L* began with TTG and *ND5* terminated with truncated T stop codon, while all other PCGs started with the standard ATN codons and terminated with the TAN codon.

**Figure 3:**
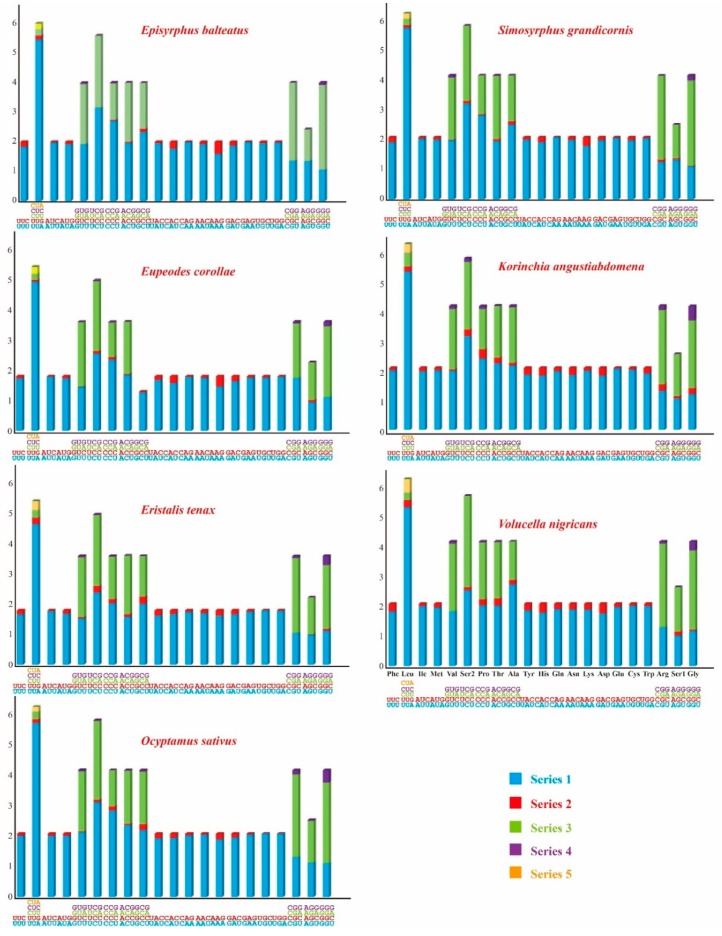
Relative synonymous codon usage (RSCU) in the mitogenomes of seven Syrphidae. The stop codon is not given. The different colors in the column chart represent the codon families corresponding to below amino acids, and the seven Syrphidae use consistent colors to represent the same codon families.

**Figure 4:**
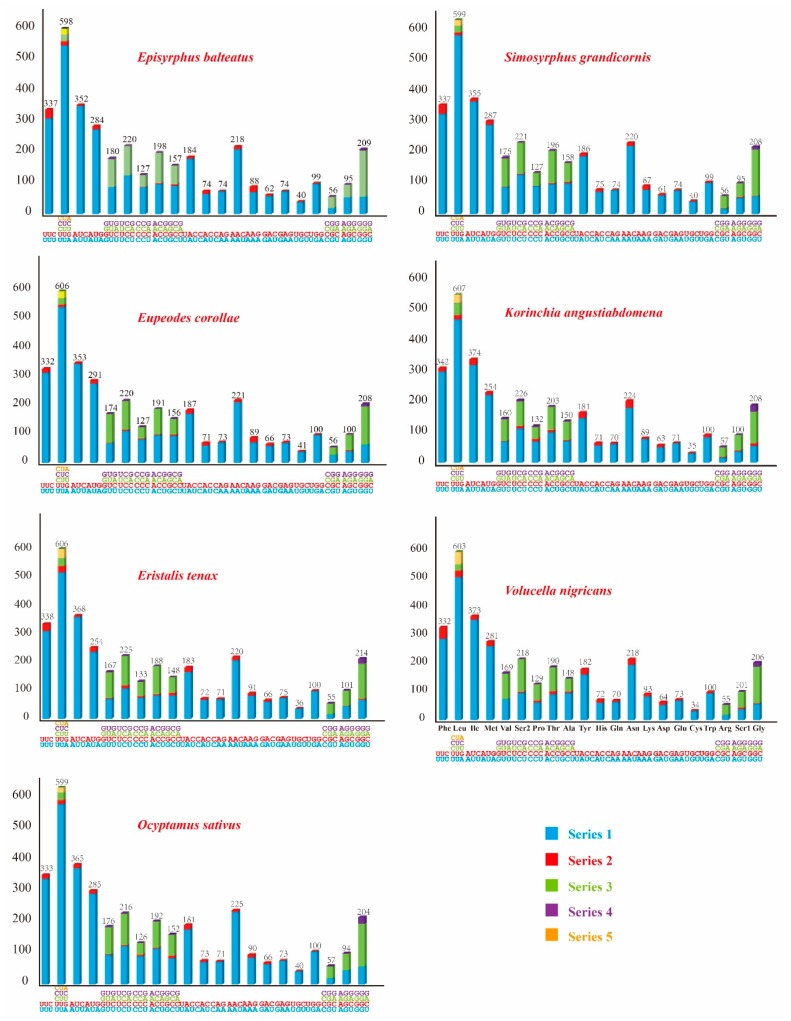
Codon distributions of seven Syrphidae mitochondrial genomes. The stop codon is not given. Numbers on the *y*-axis refer to the total number of codons. Codon families are shown on the *x*-axis.

A + T bias was also reflected in the relative codon usage by the PCGs. After excluding the stop codons, the relative synonymous codon usage (RSCU) was calculated and is summarized in Figure 3. We determined the behavior of the codon families in the PCGs (Figure 4), which showed that the codon usage was very similar in the Syrphidae, where the four most frequently used codons were Leu (598 in *E. balteatus* to 607 in *K. angustiabdomena*), Ile (352 in *E. balteatus* to 374 in *K. angustiabdomena*), Met (254 in *K. angustiabdomena* and *E. tenax* to 291 in *E. corollae*) and Phe (332 in *V. nigricans* and *E. corollae* to 342 in *K. angustiabdomena*).

### 3.3. tRNAs and rRNAs

**Figure 5:**
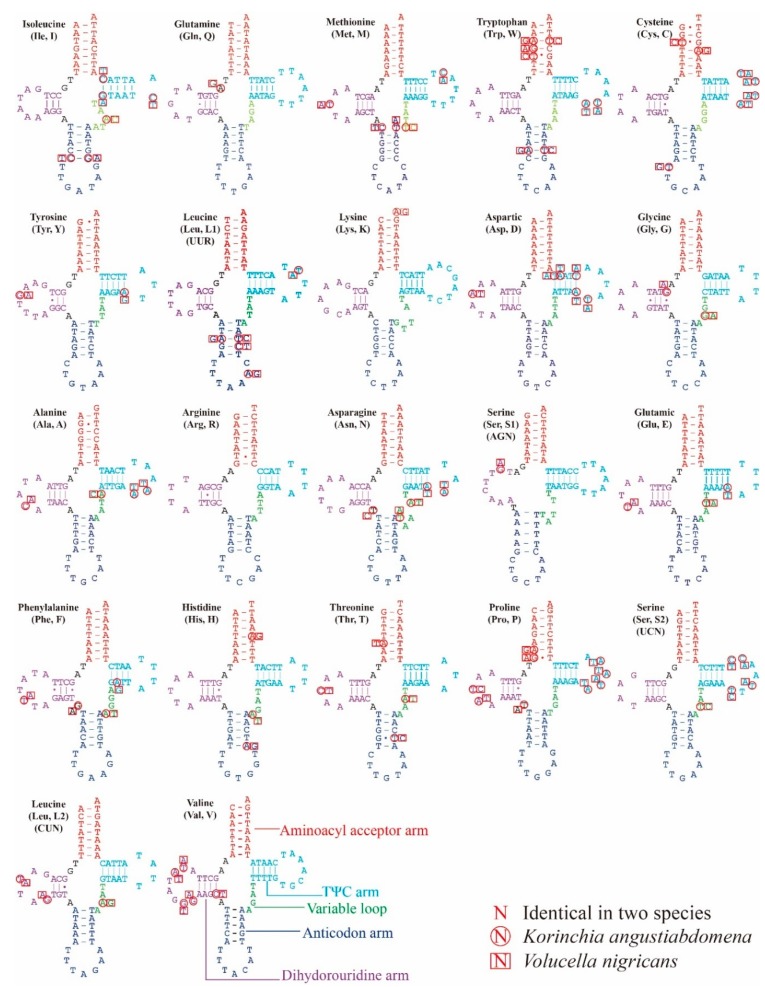
Predicted secondary structures of the 22 tRNAs in *Korinchia angustiabdomena* and *Volucella nigricans*. Dashes (–) indicate Watson–Crick base pairing and dots (.) indicate G-U base pairing.

All tRNA sequences in the two newly sequenced Syrphidae mitogenomes were determined using tRNA scan-SE or ARWEN. Most tRNAs could be folded into the typical clover-leaf structure (Figure 5), while *tRNA-Ser* (AGN) lacked a DHU arm, as has been observed in other metazoan mitogenomes [39]. The combined length of all tRNAs was 1471 bp in *K. angustiabdomena* and 1475 bp in *V. nigricans*, which are medium sized when compared with the mitogenomes of other Syrphidae for which total tRNA size ranges from 1471 bp to 1479 bp (*E. corollae* and *E. tenax* [14]). Besides the classic A-U and C-G pairs in the secondary structure, there are 18 and 15 base pairings in *K. angustiabdomena* and *V. nigricans*, respectively. Four and nine other mismatched base pairs (U-U and C-U) were also founded in the arm.

There were two rRNA genes, a 1338-bp *16S* rRNA gene and a 787 bp/186 bp *12S* rRNA genes in *K. angustiabdomena* and *V. nigricans*, respectively. Among the seven Syrphidae mitogenomes, the length of the *16S* rRNA genes range from 1,314 bp (*O. sativus* [23]) to 1,340 bp (*E. tenax*), and the length of *12S* rRNAs are 778 bp (*O. sativus* [23]) to 804 bp (*S. grandicornis* and *E. balteatus* [14,20]), with mean A + T contents of 84.5% and 83%, respectively (Table S6). Both rRNA genes were located on the N strand. Unlike PCGs with functional annotation features, it is difficult to determine rRNA gene boundaries [40,41]. Therefore, the boundaries of flanking genes were used by assuming no overlapping or gaps between adjacent genes, as in the inferred insect mitogenome pattern. The *16S* rRNA subunit was located between *tRNA-L2* (CUN) and *tRNA-V*, while the *12S* rRNA gene was between *tRNA-V* and the control region.

### 3.4. Non-Coding Region

Both newly sequenced mitogenomes had gene overlap, and each single overlap ranged from 1 to 9 bp. *K. angustiabdomena* had a total of 34 bp in overlaps between nine gene junctions, while *V. nigricans* had 38 bp overlaps between 11 gene junctions (Tables S4 and S5). Excluding the control region, there were 19 and 13 intergenic spacers of total 179 and 143 bp non-coding bases in *K. angustiabdomena* and *V. nigricans*, respectively. The longest intergenic spacers in the mitogenomes are between *tRNA-E* and *tRNA-F* (20 bp) in *K. angustiabdomena* and between *tRNA-Y* and *COX1* (34 bp) in *V. nigricans*, respectively.

The putative control region between *12S* rRNA and *tRNA-I* was the most variable region in the whole mitogenome. In *K. angustiabdomena*, the full control region measured 1526 bp in length with an A + T content of 94.4%, and a 119 bp repeat unit repeated four times within control region. In *V. nigricans*, the control region was 843 bp in length with an A + T content of 94.4%, and a 74 bp repeat unit repeated twice.

### 3.5. Phylogenetic Relationship

**Figure 6:**
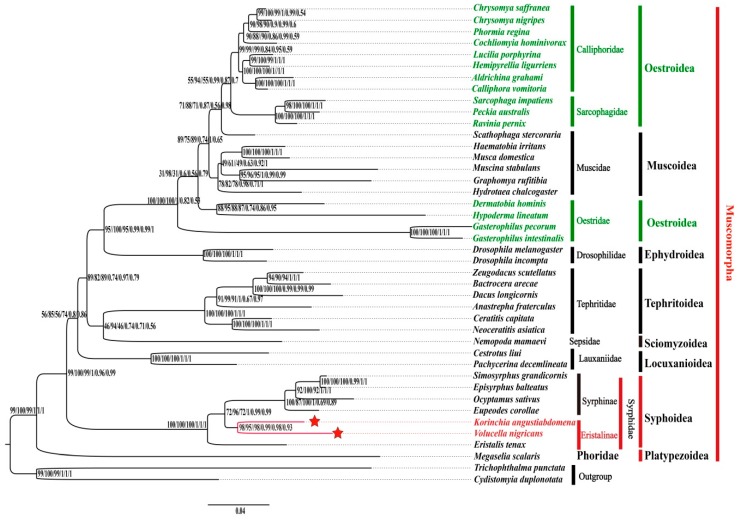
Phylogenetic relationships for Syrphidae based on the AA/PCG12RNA/PCGRNA datasets inferred from iq-tree and PhyloBayes. Numbers on branches are Bootstrap values and Bayesian posterior probabilities (PP) (ML-AA; ML-PCG12RNA; ML-PCGRNA; PB-AA; PB-PCG12RNA and PB-PCGRNA, respectively).

The phylogeny of Syrphidae and relative groups in the present study was reconstructed based on the three above datasets containing 40 Muscomorpha species and 2 outgroup species using the methods of Maximum likelihood (ML) and PhyloBayes (PB), totally six trees with strict similarity of topologies (Figure 6) were generated. Comparing with these six trees, the bootstrap probabilities and Bayesian posterior probabilities based on the dataset of PCG12RNA are higher than the other two datasets.

As for the phylogenetic relationship, Syphinae and Eristalinae grouped into one clade that belongs to Syrphidae, and they had a stable sister relationship with other ingroup species, except Phoridae. Phoridae was sister to all other species of Muscromorpha, which is congruent with previous studies [14,42,43]. The seven genera of Syrphidae were clustered as *Eristalis* + ((*Korimchia* + *Volucella*) + ((*Eupeodes* + (*Ocyptamus* + (*Simosyrphus* + *Episyrphus*))))), which differed slightly from the conclusions of Pu et al. (*Ocyptamus* + (*Eupeodes* + (*Episyrphus* + *Simosyrphus*))) [14]. In our study, the Oestroidea (Calliphoridae, Sarcophagidae and Oestridae) consisted of 15 selected species unexpectedly found to be paraphyletic, which seemingly does not agree with the result by Pu et al. [14], but it is very necessary to further examine if more abundant molecular data (more species, more mitogenomes, or more longer sequences, etc.) increased. The phylogenetic relationship of Muscomorpha showed that (((((Oestroidea + Muscoidea) + Ephydroidea) + (Tephritoidea + Sciomyzoidea)) + Locuxanioidea) + Syphoidea) + Platypezoidea.

## 4. Conclusions

Consistent with previous observations of Syrphidae species, the mitogenome sequences of *K. angustiabdomena* and *V. nigricans* were highly conserved in gene order, gene content, gene size, base composition, codon usage of PCGs, and tRNA secondary structures. Variation in the length of complete mitogenomes is mostly due to the length of the control region, which ranges from 320 bp (*E. corollae* [14]) to 1526 bp (*K. angustiabdomena*, this study).

ML and PB analyses showed that each family in the tree formed a monophyletic clade within Muscomorpha, but the superfamily Oestroidea (Calliphoridae, Sarcophagidae and Oestridae) was unexpectedly found to be a paraphyletic group in this study. The seven genera of Syrphidae were clustered as *Eristalis* + ((*Korimchia* + *Volucella*) + ((*Eupeodes* + (*Ocyptamus* + (*Simosyrphus* + *Episyrphus*))))).

## Figures and Tables

**Table 1:** Primers used in this study.

Primer Name	Sequences 5′-3′	References
1	COX1F: ACAAATCATAAAGATGG	[25]
	COX1R: TGATTTTAGGTCACCCTGA	
2	12SF: TACTATGTTACGACTTAT	[26]
	12SR: AAACTAGGATTAGATACCC	

**Table 2:** AT- and GC-skew in different regions of seven sequenced Syrphidae mitogenomes.

Species	Whole Mitochondrial Genome							PCGs						
	Length	A	T	G	C	AT Skew	GC Skew	Length	A	T	G	C	AT Skew	GC Skew
*Korinchia angustiabdomena*	16,473	40.8	39.6	7.9	11.8	0.01	−0.20	11,188	33.1	44.5	11.1	11.3	−0.15	−0.01
*Volucella nigricans*	15,724	42.1	37.8	7.9	12.2	0.05	−0.21	11,170	34.1	43.8	11.1	11.0	−0.12	0.00
*Ocyptamus sativus*	15,214 (Partial)							11,193	33.9	45.1	11.0	10.0	−0.14	0.05
*Simosyrphus grandicornis*	16,141	40.3	40.6	8.3	10.9	0.00	−0.14	11,229	34.0	44.9	10.9	10.3	−0.14	0.03
*Episyrphus balteatus*	16,175	40.3	40.5	8.4	10.8	0.00	−0.13	11,217	33.9	44.9	11.0	10.2	−0.14	0.04
*Eupeodes corollae*	15,326	40.5	39.7	8.6	11.2	0.01	−0.13	11,244	34.0	44.8	11.0	10.1	−0.14	0.04
*Eristalis tenax*	15,996	41.0	39.3	8.2	11.5	0.02	−0.17	11,170	33.7	44.4	11.2	10.7	−0.14	0.03
**Species**	**RNAs**							**Control Region**						
	**Length**	**A**	**T**	**G**	**C**	**AT Skew**	**GC Skew**	**Length**	**A**	**T**	**G**	**C**	**AT Skew**	**GC Skew**
*Korinchia angustiabdomena*	3606	40.2	41.9	11.1	6.8	−0.02	0.24	1526	47.6	46.8	1.5	4.1	0.01	−0.47
*Volucella nigricans*	3599	40.3	41.6	11.1	6.9	−0.02	0.23	843	48.8	46.6	1.7	3.0	0.02	−0.28
*Ocyptamus sativus*	3570	41.0	41.7	10.6	6.6	−0.01	0.23	92 (Partial)						
*Simosyrphus grandicornis*	3618	41.4	41.5	10.5	6.6	0.00	0.23	1127	44.2	47.6	3.6	4.5	−0.04	−0.11
*Episyrphus balteatus*	3617	41.4	41.4	10.6	6.7	0.00	0.23	1129	43.7	47.8	3.6	4.9	−0.05	−0.15
*Eupeodes corollae*	3608	40.9	41.6	10.8	6.7	−0.01	0.23	320	46.9	45.3	2.5	5.3	0.02	−0.36
*Eristalis tenax*	3611	40.5	41.4	11.2	7.0	−0.01	0.23	1073	48.5	47.7	1.7	2.1	0.01	−0.12

## Supplementary Materials

The following are available online at https://www.mdpi.com/2073-4425/10/8/563/s1, Figure S1: An agarose gel showing the bands of PCR fragments of *COX1* (1–2) and *12s* rRNA (3–4). M: Marker; 1, 3: *Korinchia angustiabdomena*; 2, 4: *Volucella nigricans*, Table S1: Collection information of specimens in the present study, Table S2: List of mitochondrial genomes used for the phylogenetic analysis in this study, Table S3: Partition strategies and evolutionary models used in ML analysis, Table S4: Organization of the *Korinchia angustiabdomena* mitogenome, Table S5: Organization of the *Volucella nigricans* mitogenome, Table S6: Nucleotide compositions, AT- and GC-skew in rRNAs of sequenced Syphidae mitochondrial genomes.
