# Supplementary material for: Characterization and Phylogenetic Implications of the Complete Mitochondrial Genome of Syrphidae
Source: Genes (Basel). 2019 Jul 25;10(8):563. doi: 10.3390/genes10080563 (PMC6723807; doi:10.3390/genes10080563)
Supplement: Supplementary file 1 [file genes-10-00563-s001.pdf]

Table S1. Collection information of specimens in the present study.

Name	Locality	Time	Collecter
V. nigricans	Huayang ancient town, hanzhongyang, shaanxi	23, July 2018	Hu Li
K. angustiabdomena	Huayang ancient town, hanzhongyang, shaanxi	25, July 2018	Hu Li

Note: *V. nigricans* indicates *Volucella nigricans* and *K. angustiabdomena* indicates *Korinchia angustiabdomena*

Table S2. List of mitochondrial genomes used for the phylogenetic analysis in this study.

	family	Species	NCBI No.	References
Ingroups	Phoridae	*Megaselia scalaris*	NC_023794	[1]
	Tephritidae	*Anastrepha fraterculus*	NC_034912	[2]
		Bactrocera arecae	NC_028327	[3]
		Ceratitis capitata	AJ242872	[4]
		Dacus longicornis	NC_032690	[5]
		Neoceratitis asiatica	MF434829	[6]
		Zeugodacus scutellatus	MF358969	[7]
	Drosophilidae	*Drosophila melanogaster*	NC_024511	[8]
		Drosophila incompta	NC_025936	[9]
	Sepsidae	*Nemopoda mamaevi*	NC_026866	[10]
	Muscidae	*Musca domestica*	NC_024855	[11]
		Haematobia irritans	NC_007102	
		Muscina stabulans	NC_026292	
		Hydrotaea chalcogaster	NC_041089	[12]
		Graphomya rufitibia	NC_038210	[13]
		Scathophaga stercoraria	NC_024856	[14]
	Calliphoridae	*Chrysomya nigripes*	NC_028412	
		Aldrichina grahami	NC_026996	
		Cochliomyia hominivorax	NC_002660	[15]
		Calliphora vomitoria	NC_028411	
		Phormia regina	NC_026668	[16]
		Lucilia porphyrina	NC_019637	
		Hemipyrellia ligurriens	NC_019638	[17]
		Chrysomya saffranea	NC_019635	
	Oestridae	*Dermatobia hominis*	NC_006378	
		Gasterophilus pecorum	NC_029812	[18]
		Gasterophilus intestinalis	NC_029834	[19]
		Hypoderma lineatum	NC_013932	[20]
	Sarcophagidae	*Sarcophaga impatiens*	NC_017605	[21]
		Ravinia pernix	NC_026196	
		Peckia australis	NC_041078	[22]
	Lauxaniidae	*Pachycerina decemlineata*	NC_034923	[23]
		Cestrotus liui	NC_034922	
	Syrphidae	*Ocyptamus sativus*	KT272862	[24]
		Simosyrphus grandicornis	NC_008754	[25]
		Episyrphus balteatus	NC_036481	[26]
		Eupeodes corollae	NC_036482	
		Volucella nigricans	MK870079	This study
		Korinchia angustiabdomena	MK870078	This study
		Eristalis tenax	NC_041143	
Outgroups	Tabanidae	*Cydistomyia duplonotata*	NC_008756	

Nemestrinidae	*Trichophthalma punctata*	NC_008755	[27]

[1] Zhong M, Wang X, Liu Q, et al. The complete mitochondrial genome of the scuttle fly, Megaselia scalaris (Diptera: Phoridae) [J]. Mitochondrial DNA Part A, 2016, 27(1): 182-184.

[2] Isaza J P, Alzate J F, Canal N A. Complete mitochondrial genome of the Andean morphotype of Anastrepha fraterculus (Wiedemann) (Diptera: Tephritidae)[J]. Mitochondrial DNA Part B, 2017, 2(1): 210-211.

[3] Yong H S, Song S L, Lim P E, et al. Complete mitochondrial genome of Bactrocera arecae (Insecta: Tephritidae) by next-generation sequencing and molecular phylogeny of Dacini tribe[J]. Scientific reports, 2015, 5: 15155.

[4] Spanos, L., Koutroumbas, G., Kotsyfakis, M., & Louis, C. (2000). The mitochondrial genome of the Mediterranean fruit fly, Ceratitis capitata. Insect Molecular Biology, 9(2), 139-144.

[5] Jiang, F., Pan, X., Li, X., Yu, Y., Zhang, J., Jiang, H., ... & Zhu, S. (2016). The first complete mitochondrial genome of Dacus longicornis (Diptera: Tephritidae) using next-generation sequencing and mitochondrial genome phylogeny of Dacini tribe. Scientific reports, 6, 36426.

[6] Su Y, Zhang Y, Feng S, et al. The mitochondrial genome of the wolfberry fruit fly, Neoceratitis asiatica (Becker)(Diptera: Tephritidae) and the phylogeny of Neoceratitis Hendel genus[J]. Scientific reports, 2017, 7(1): 16612.

[7] Liu J H, Jia P F, Liu L L, et al. Complete mitochondrial genome of stripped fruit fly, Bactrocera (Zeugodacus) scutellata (Diptera: Tephritidae) from Anshun, Southwest China[J]. Mitochondrial DNA Part B, 2017, 2(2): 387-388.

[8] Wolff J N, Camus M F, Clancy D J, et al. Complete mitochondrial genome sequences of thirteen globally sourced strains of fruit fly (Drosophila melanogaster) form a powerful model for mitochondrial research[J]. Mitochondrial DNA Part A, 2016, 27(6): 4672-4674.

[9] De Ré F C, Wallau G L, Robe L J, et al. Characterization of the complete mitochondrial genome of flower-breeding Drosophila incompta (Diptera, Drosophilidae)[J]. Genetica, 2014, 142(6): 525-535.

[10] Li X, Ding S, Cameron S L, et al. The First Mitochondrial genome of the sepsid fly Nemopoda mamaevi Ozerov, 1997 (Diptera: Sciomyzoidea: Sepsidae), with mitochondrial genome phylogeny of Cyclorrhapha[J]. PloS one, 2015, 10(3): e0123594.

[11] Li X, Wang Y, Su S, et al. The complete mitochondrial genomes of Musca domestica and Scathophaga stercoraria (Diptera: Muscoidea: Muscidae and Scathophagidae)[J]. Mitochondrial DNA Part A, 2016, 27(2): 1435-1436.

[12] Zhang J, Deng S. The complete mitochondrial genome of Hydrotaea (Ophyra) chalcogaster (Diptera: Muscidae)[J]. Mitochondrial DNA Part B, 2018, 3(2): 959-959.

[13] Chen W, Shang Y, Ren L, et al. The complete mitochondrial genome of Graphomya rufitibia (Diptera: Muscidae)[J]. Mitochondrial DNA Part B, 2018, 3(1): 403-404.

[14] Li X, Wang Y, Su S, et al. The complete mitochondrial genomes of Musca domestica and Scathophaga stercoraria (Diptera: Muscoidea: Muscidae and Scathophagidae)[J]. Mitochondrial DNA Part A, 2016, 27(2): 1435-1436.

[15] Lessinger A C, Martins Junqueira A C, Lemos T A, et al. The mitochondrial genome of the primary screwworm fly Cochliomyia hominivorax (Diptera: Calliphoridae)[J]. Insect Molecular Biology, 2000, 9(5): 521-529.
[16] Ramakodi M P, Singh B, Wells J D, et al. A 454 sequencing approach to dipteran mitochondrial genome research[J]. Genomics, 2015, 105(1): 53-60.
[17] Nelson L A, Lambkin C L, Batterham P, et al. Beyond barcoding: A mitochondrial genomics approach to molecular phylogenetics and diagnostics of blowflies (Diptera: Calliphoridae)[J]. Gene, 2012, 511(2): 131-142.
[18] Zhang D, Yan L, Zhang M, et al. Phylogenetic inference of calyptrates, with the first mitogenomes for Gasterophilinae (Diptera: Oestridae) and Paramacronychiinae (Diptera: Sarcophagidae)[J]. International Journal of Biological Sciences, 2016, 12(5):489-504. doi:10.7150/ijbs.12148
[19] Gao D Z, Liu G H, Song H Q, et al. The complete mitochondrial genome of Gasterophilus intestinalis, the first representative of the family Gasterophilidae[J]. Parasitology research, 2016, 115(7): 2573-2579.
[20] Weigl S, Testini G, Parisi A, et al. The mitochondrial genome of the common cattle grub, Hypoderma lineatum[J]. Medical and veterinary entomology, 2010, 24(3): 329-335.
[21] Nelson L A, Cameron S L, Yeates D K. The complete mitochondrial genome of the flesh fly, Sarcophaga impatiens Walker (Diptera: Sarcophagidae) [J]. Mitochondrial DNA, 2012, 23(1): 42-43.
[22] Faccin S, Carmo A O, Thyssen P J, et al. Complete mitochondrial genomes from three species of the genus Peckia (Sarcophagidae) with forensic entomology interest[J]. Mitochondrial DNA Part B, 2019, 4(1): 220-221.
[23] Li X, Li W, Ding S, et al. Mitochondrial genomes provide insights into the phylogeny of Lauxanioidea (Diptera: Cyclorrhapha) [J]. International journal of molecular sciences, 2017, 18(4): 773.
[24] Junqueira A C M, Azeredo-Espin A M L, Paulo D F, et al. Large-scale mitogenomics enables insights into Schizophora (Diptera) radiation and population diversity[J]. Scientific reports, 2016, 6: 21762.
[25] Cameron S L, Lambkin C L, Barker S C, et al. A mitochondrial genome phylogeny of Diptera: whole genome sequence data accurately resolve relationships over broad timescales with high precision[J]. Systematic Entomology, 2007, 32(1): 40-59.
[26] Pu D, Liu H, Gong Y, et al. Mitochondrial genomes of the hoverflies Episyrphus balteatus and Eupeodes corollae (Diptera: Syrphidae), with a phylogenetic analysis of Muscomorpha[J]. Scientific reports, 2017, 7: 44300.
[27] Cameron S L, Lambkin C L, Barker S C, et al. A mitochondrial genome phylogeny of Diptera: whole genome sequence data accurately resolve relationships over broad timescales with high precision[J]. Systematic Entomology, 2007, 32(1): 40-59.

Table S3. Partition strategies and evolutionary models used in ML analysis.

Dataset	Subset	Best Model	Score	Partition names
PCG12RNA 7 partitions	1	TIM2+F+I+G4	31812.155	cox1_pos1, cox2_pos1, cox3_pos1, cob_pos1, atp6_pos1
	2	TVM+F+R3	12536.897	atp6_pos2, cob_pos2, cox1_pos2, cox2_pos2, cox3_pos2
	3	GTR+F+I+G4	22812.410	ad6_pos1, nad3_pos1, atp8_pos1, nad2_pos1
	4	TVM+F+I+G4	12633.906	nad6_pos2, atp8_pos2, nad2_pos2, nad3_pos2
	5	GTR+F+I+G4	31527.481	nad4_pos1, nad5_pos1, nad1_pos1, nad4l_pos1
	6	GTR+F+R4	18576.627	nad4_pos2, nad4l_pos2, nad5_pos2, nad1_pos2
	7	GTR+F+R4	44215.658	rrnS, rrnL
PCGRNA 10 partitions	1	GTR+F+I+G4	31924.912	cox1_pos1, cox3_pos1, cox2_pos1, cob_pos1, atp6_pos1
	2	TVM+F+R3	12712.388	atp6_pos2, cob_pos2, cox1_pos2, cox3_pos2, cox2_pos2
	3	TPM3u+F+R5	100584.070	cob_pos3, nad3_pos3, cox3_pos3, cox2_pos3, nad6_pos3, atp8_pos3, cox1_pos3, atp6_pos3
	4	GTR+F+I+G4	22932.849	nad6_pos1, nad3_pos1, atp8_pos1, nad2_pos1
	5	TVM+F+I+G4	12841.048	atp8_pos2, nad2_pos2, nad3_pos2, nad6_pos2
	6	GTR+F+I+G4	31849.066	nad4_pos1, nad5_pos1, nad1_pos1, nad4l_pos1
	7	GTR+F+R4	18823.436	nad4_pos2, nad4l_pos2, nad1_pos2, nad5_pos2
	8	TIM3+F+R5	69354.080	nad4l_pos3, nad5_pos3, nad4_pos3, nad1_pos3
	9	TPM3u+F+I+G4	18334.308	nad2_pos3
	10	GTR+F+R4	44519.431	rrnL, rrnS
AA 4 partitions	1	mtART+R4	13560.661	atp6, nad1
	2	mtMet+F+R5	33559.415	nad3, atp8, nad6, nad2
	3	mtZOA+R4	29086.044	cox2, cox3, cox1, cob
	4	mtInv+R5	41596.083	nad5, nad4, nad4l

Table S4. Organization of the *Korinchia angustiabdomena* mitogenome.

Name	Direction	Location	Size(bp)	Anti / Start/ Stop codon	IN
tRNA-I	F	1-66	66	30-32 GAT	
tRNA-Q	R	64-132	69	102-100 TTG	-3
tRNA-M	F	140-208	69	170-172 CAT	7
ND2	F	209-1231	1023	ATT/TAA	0
tRNA-W	F	1230-1297	68	1260-1262 TCA	-2
tRNA-C	R	1290-1354	65	1325-1323 GCA	-8
tRNA-Y	R	1371-1436	66	1405-1403 GTA	16
COX1	F	1447-2973	1527	ATA/TAA	10
tRNA-L1	F	2969-3033	65	2998-3000 TAA	-5
COX2	F	3037-3720	684	ATG/TAA	3
tRNA-K	F	3722-3792	71	3752-3754 CTT	1
tRNA-D	F	3871-3938	68	3902-3904 GTC	78
ATP8	F	3939-4100	162	ATT/TAA	0
ATP6	F	4097-4771	675	ATA/TAA	-4
COX3	F	4771-5559	789	ATG/TAA	-1
tRNA-G	F	5566-5630	65	5595-5597 TCC	6
ND3	F	5631-5984	354	ATT/TAA	0
tRNA-A	F	5990-6056	67	6020-6022 TGC	5
tRNA-R	F	6056-6118	63	6085-6087 TCG	-1
tRNA-N	F	6120-6186	67	6151-6153 GTT	1
tRNA-S1	F	6187-6253	67	6212-6214 GCT	0
tRNA-E	F	6255-6320	66	6285-6287 TTC	1
tRNA-F	R	6341-6406	66	6374-6372 GAA	20
ND5	R	6407-8141	1735	ATT/T--	0
tRNA-H	R	8139-8204	66	8174-8172 GTG	-3
ND4	R	8209-9549	1341	ATG/TAA	4
ND4L	R	9543-9839	297	ATG/TAA	-7
tRNA-T	F	9842-9906	65	9872-9874 TGT	2
tRNA-P	R	9907-9973	67	9943-9941 TGG	0
ND6	F	9976-10500	525	ATT/TAA	2
Cytb	F	10504-11640	1137	ATG/TAA	3
tRNA-S2	F	11643-11710	68	11672-11674 TGA	2
ND1	R	11727-12665	939	ATA/TAG	16
tRNA-L2	R	12676-12740	65	12711-12709 TAG	10
16S	R	12741-14078	1338		0
tRNA-V	R	14079-14150	72	14117-14115 TAC	0
12S	R	14151-14947	797		0
CR		14948-16473	1526		0

NOTE: IN (Intergenic nucleotides): indicates gap nucleotides (positive value) or overlapped nucleotides (negative value) between two adjacent genes. CR means Control Region.

Table S5. Organization of the *Volucella nigricans* mitogenome.

Name	Direction	Location	Size(bp)	Anti / Start/ Stop codon	IN
tRNA-I	F	1-66	66	30-32 GAT	0
tRNA-Q	R	81-149	69	119-117 TTG	14
tRNA-M	F	149-217	69	179-181 CAT	-1
ND2	F	218-1237	1,020	ATT/TAA	0
tRNA-W	F	1235-1304	70	1266-1268 TCA	-3
tRNA-C	R	1296-1360	65	1331-1329 GCA	-9
tRNA-Y	R	1372-1437	66	1406-1404 GTA	11
COX1	F	1472-2977	1,506	ATT/TAA	34
tRNA-L1	F	2973-3038	66	3002-3004 TAA	-5
COX2	F	3048-3731	684	ATG/TAA	9
tRNA-K	F	3732-3802	71	3762-3764 CTT	0
tRNA-D	F	3803-3869	67	3834-3836 GTC	0
ATP8	F	3870-4031	162	ATT/TAA	0
ATP6	F	4028-4702	675	ATA/TAA	-4
COX3	F	4702-5490	789	ATG/TAA	-1
tRNA-G	F	5502-5568	67	5531-5533 TCC	11
ND3	F	5566-5922	357	ATA/TAA	-3
tRNA-A	F	5925-5991	67	5955-5957 TGC	2
tRNA-R	F	5991-6053	63	6020-6022 TCG	-1
tRNA-N	F	6060-6126	67	6090-6092 GTT	6
tRNA-S1	F	6127-6193	67	6152-6154 GCT	0
tRNA-E	F	6195-6259	65	6225-6227 TTC	1
tRNA-F	R	6281-6347	67	6315-6313 GAA	21
ND5	R	6348-8085	1,738	ATT/T--	0
tRNA-H	R	8083-8148	66	8115-8113 GTG	-3
ND4	R	8149-9489	1,341	ATG/TAA	0
ND4L	R	9483-9779	297	ATG/TAA	-7
tRNA-T	F	9782-9847	66	9812-9814 TGT	2
tRNA-P	R	9848-9913	66	9883-9881 TGG	0
ND6	F	9916-10440	525	ATT/TAA	2
Cytb	F	10440-11576	1,137	ATG/TAA	-1
tRNA-S2	F	11584-11651	68	11613-11615 TGA	7
ND1	R	11672-12610	939	ATA/TAA	20
tRNA-L2	R	12621-12685	65	12656-12654 TAG	10
16S	R	12686-14023	1,338		0
tRNA-V	R	14024-14095	72	14062-14060 TAC	0
12S	R	14096-14881	786		0
CR		14882-15724	843		0

NOTE: IN (Intergenic nucleotides): indicates gap nucleotides (positive value) or overlapped nucleotides (negative value) between two adjacent genes. CR means Control Region.

Table S6. Nucleotide compositions, AT- and GC-skew in rRNAs of sequenced Syphidae mitochondrial genomes.

Species	16S								12S							
	Length	A	T	G	C	AT	AT Skew	GC Skew	Length	A	T	G	C	AT	AT Skew	GC Skew
Korinchia angustiabdomena	1338	43.3	40.8	5.4	10.5	84.2	0.03	-0.32	787	43	39.4	5.9	11.7	82.4	0.04	-0.33
Volucella nigricans	1338	43.7	40.7	5.4	10.2	84.5	0.04	-0.31	786	42	40.1	6.1	11.8	82.1	0.02	-0.32
Ocyptamus sativus	1314	42.7	41.7	5.5	10.1	84.4	0.01	-0.29	778	41.8	41	6.2	11.1	82.8	0.01	-0.28
Simosyrphus grandicornis	1339	43	42	5.3	9.7	85	0.01	-0.29	804	42.2	41.7	5.8	10.3	83.8	0.01	-0.28
Episyrphus balteatus	1338	42.8	41.8	5.5	9.9	84.6	0.01	-0.29	804	42.2	41.8	5.8	10.2	84	0.00	-0.28
Eupeodes corollae	1334	43.4	41.4	5.3	9.9	84.8	0.02	-0.30	795	42.3	40.9	5.9	10.9	83.1	0.02	-0.30
Eristalis tenax	1340	42.6	41.1	5.6	10.7	83.7	0.02	-0.31	792	42.2	40.5	5.9	11.4	82.7	0.02	-0.32
mean	1334	43.1	41.4	5.4	10.1	84.5	0.02	-0.30	792	42.2	40.8	5.9	11.1	83.0	0.02	-0.30

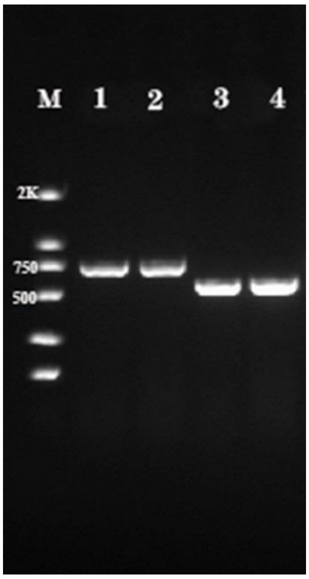

Figure S1. An agarose gel showing the bands of PCR fragments of *COX1* (1–2) and *12s* rRNA (3–4). M: Marker; 1, 3: *Korinchia angustiabdomena*; 2, 4: *Volucella nigricans*.
